# Associations of Cigarette Smoking and Alcohol Consumption With Metabolic Syndrome in a Male Chinese Population: A Cross-Sectional Study

**DOI:** 10.2188/jea.JE20130112

**Published:** 2014-09-05

**Authors:** Min Yu, Chun-Xiao Xu, Hong-Hong Zhu, Ru-Ying Hu, Jie Zhang, Hao Wang, Qin-Fang He, Dan-Ting Su, Min Zhao, Li-Xin Wang, Wei-Wei Gong, Jin Pan, Le Fang, Zhen Ye

**Affiliations:** 1 Department of Chronic Non-Communicable Diseases Control and Prevention, Zhejiang Provincial Center for Disease Control and Prevention, Hangzhou, Zhejiang, P. R. China; 2 Department of Public Health, College of Health and Human Services, Western Kentucky University, Bowling Green, Kentucky, USA; 3 General Office, Health Bureau of Zhejiang Province, Hangzhou, Zhejiang, P. R. China

**Keywords:** cigarette smoking, alcohol consumption, metabolic syndrome, Chinese man, interaction

## Abstract

**Background:**

Whether cigarette smoking and alcohol consumption are associated with the risk of metabolic syndrome (MetS) remains controversial. This study investigated the associations of cigarette smoking and alcohol consumption with MetS in a male population in China.

**Methods:**

We conducted a cross-sectional study. A questionnaire was used to collect data on cigarette smoking, alcohol consumption, MetS status, and other related information from 8169 men aged 19–97 years. Logistic regression was used to estimate the odds ratios (ORs) and 95% confidence intervals (CIs) for the associations between smoking and alcohol consumption and the risk of MetS.

**Results:**

The prevalence of MetS was 15.2% in the study population. Proportions of current smokers and drinkers were 48.2% and 46.5%, respectively. Adjusted OR of MetS was 1.34 (95% CI, 1.01–1.79) among smokers who smoked ≥40 cigarettes/day compared with nonsmokers and 1.22 (95% CI 1.03–1.46) for those who consumed 0.1–99 grams of alcohol/day compared with nondrinkers. Adjusted OR was 2.32 (95% CI 1.45–3.73) among ex-drinkers who never smoked, 1.98 (95% CI 1.35–2.91) among ex-drinkers who were current smokers, and 1.34 (95% CI 1.08–1.68) among current drinkers who never smoked compared with those who neither smoked nor drank. There was a significant interaction between smoking and drinking alcohol on MetS (*P* for interaction is 0.001).

**Conclusions:**

Our study indicated that smoking and drinking is associated with higher prevalence of MetS. Interactions between smoking and drinking on the risk of MetS in men in China may also exist. Our findings need to be confirmed in future case-control or cohort studies.

## INTRODUCTION

Metabolic syndrome (MetS) is a combination of medical disorders including abdominal obesity, hypertension, dyslipidemia, and glucose intolerance, that, when occurring together, increase the risk of developing cardiovascular disease and type 2 diabetes mellitus.^1^ People with MetS are three times as likely to suffer from heart attack or stroke as those without the condition.^1^ MetS is considered to be a risk factor for type 2 diabetes mellitus and cardiovascular disease.^2^ It is estimated that around 20%–25% of the world’s adult population has MetS.^1^ The prevalence of MetS is currently around 16.5% in China and is rising nationwide.^3^

Unhealthy lifestyles such as cigarette smoking and alcohol consumption have been causing many health problems in the past decades. Evidence has shown that smoking can increase blood pressure, waist circumference, and triglyceride (TG) levels, as well as reduce high density lipoprotein cholesterol (HDL-C) levels^4^^,^^5^ and insulin sensitivity or induce insulin resistance.^6^ Smoking is considered a major risk factor for metabolic and cardiovascular diseases.^7^

A large population-based study in the United States reported that mild-to-moderate alcohol consumption was associated with a favorable influence on lipids, waist circumference, and fasting insulin compared with nondrinkers.^8^ Increased alcohol consumption, however, has also been reported to be associated with hypertension.^9^ Furthermore, insulin resistance, which is the key pathophysiology of MetS, has been reported to relate to alcohol consumption in a U-shaped manner.^10^

These findings add complexity to of the relationships between smoking and alcohol consumption and MetS. Findings regarding the association of MetS with tobacco smoking have been controversial. Significant positive associations of smoking with MetS have been reported in Japan and Korean populations.^11^^–^^13^ A negative association of smoking with MetS, however, was reported by Onat et al^14^ in a Turkish population. As for the association between MetS and alcohol consumption, studies have also shown inconsistent results. Some studies have shown positive associations,^15^ whereas others have observed a negative associations^16^^,^^17^ or no association at all.^18^^–^^20^

Assessment of the overall associations between cigarette smoking and alcohol consumption and the development of metabolic syndrome is needed. However, only a few studies focusing on the association between cigarette smoking and/or alcohol consumption and the risk of MetS have been done in Chinese populations.^5^^,^^21^^–^^23^ Among these studies, none have explored the interactions between cigarette smoking and alcohol consumption and the risk of MetS. Assessing the influence of smoking and drinking on MetS has important public health implications since central obesity, hypertension, hyperglycemia, and hyperlipidemia are common conditions that are frequently diagnosed in the same individual. Therefore, this study investigated the associations between cigarette smoking and alcohol consumption and the prevalence of MetS and its components and also explored the interactions of cigarette smoking and alcohol consumption with MetS in a male population in China.

## METHODS

### Study design and population

We conducted a large-scale population-based cross-sectional study from 2009 to 2010 that was designed by our department to estimate the prevalence of MetS in Zhejiang Province. Multi-stage stratified cluster sampling was applied to select the study participants.^24^ In the first stage, we defined 5 groups from 91 counties in Zhejiang Province based on their economic level: Type 1 urban district (highest level), Type 2 urban districts, Type 1 rural county, Type 2 rural county, and Type 3 rural county (lowest level). Three counties were then randomly selected from each group. A total of 15 areas were included in the first stage. In the second stage, four streets were randomly selected from each selected county. In the third stage, three villages were randomly selected from each selected street. In the last stage, all households in the selected villages were divided into different clusters based on geographic location; every cluster consisted of forty households. One cluster was then randomly selected from the clusters in each village.

The field work was mainly done by the Centers for Disease Control and Prevention and Centers for Community Health Service at the county level. All participants had no diagnosed cancer or mental illness, were not receiving any medication, and were aged 18 years or older at the time of enrollment. Considering the very small proportion of smoking women (3.5% former and current smokers) in this study, the analysis was restricted to men only. A total of 8169 male adults aged from 19 to 97 years old were finally included in the analysis. Signed written consent was obtained from all participants, and the study protocol was approved by the Ethics Committee of Zhejiang Provincial Center for Disease Control and Prevention.

### Physical examination and lipid profiles

Height, weight, and waist circumference were measured with the subjects wearing light clothes and no shoes. Body mass index (BMI) was calculated as weight in kilograms divided by the square of height in meters (kg/m^2^). Blood pressure measurements and fasting blood samples were taken. Serum glucose, triglyceride, and HDL-cholesterol concentrations were analyzed after a 12-h fast using a Hitachi 7180 Autoanalyzer (Hitachi Ltd., Tokyo, Japan).

### Assessment of cigarette smoking and alcohol consumption

Each participant was asked to report their daily number of cigarettes smoked and duration of smoking. Based on daily cigarette consumption, smoking status was classified into five categories: nonsmoker, 1–9 cigarettes per day, 10–19 cigarettes per day, 20–39 cigarettes per day, and ≥40 cigarettes per day. Participants were also asked about alcohol consumption per week and the type of alcoholic beverage consumed, from among beer, white wine, red wine, yellow wine (a typical wine made from rice and popularly drunk in Southern China), and hard liquor, to estimate total alcohol intake. Overall, based on weekly consumption, alcohol intake was classified into five categories: nondrinker, 0.1–99 g per week, 100–199 g per week, 200–299 g per week, and ≥300 g per week.

### Assessment of dietary intake and physical activity

Food frequency and quantitative intake was assessed by administering a questionnaire composed questions regarding of 144 food items. According to the caloric value of each item per 50 g, daily total caloric intake was calculated.

In a categorical classification of leisure time physical activity,^25^ those who reported exercising ≥6 times per month for a mean duration of ≥30 minutes and with a mean intensity corresponding to at least vigorous walking to jogging were classified as conditioning exercisers. Those who reported not partaking in physical activity during leisure time were considered sedentary. Sedentary subjects were further asked to report the intensity of their activities that did not exceed walking intensity and those participated in <6 times a month. Other subjects were classified as occasional exercisers.

### MetS definition

The International Diabetes Federation clinical criteria were used to ascertain MetS.^26^ A person was defined as having MetS if they had central obesity plus any two of four factors including increased TG (≥1.7 mmol/L or specific treatment for TG abnormality), HDL-C (<1.03 mmol/L in males or specific treatment for HDL abnormality), increased blood pressure (systolic blood pressure ≥130 or diastolic blood pressure ≥85, or treatment of previously diagnosed hypertension), and increased fasting plasma glucose (≥5.6 mmol/L or previously diagnosed type 2 diabetes). Central obesity was assessed using a Chinese-specific cutoff in which a waist circumference ≥90 cm was considered to be obese.

### Statistical analyses

Logistic regression analyses were used to evaluate the associations between MetS and its five components and cigarette smoking and alcohol consumption by calculating adjusted odds ratios (ORs) and 95% confidence intervals (CIs). Adjusted factors included age (continuous), cigarette/alcohol consumption (categorical), daily calorie intake (continuous), and physical activity (categorical). The interaction between cigarette smoking and alcohol consumption was evaluated by logistic regression, inserting smoking+alcohol consumption (categorical) as an interaction term with nondrinker and nonsmoker as references and evaluating the linear trend using the trend test. Data were analyzed using SPSS statistical package version 19.0 (IBM, Armonk, NY, USA). A *P* value <0.05 was considered to be statistically significant. Sensitivity analyses were also conducted to explore whether the results would be affected by multiple testing, by using Bonferroni corrections where the significance was defined as *P*-value <0.0042 (0.05/12).

## RESULTS

Basic characteristics of study participants by MetS are shown in Table 1. The mean age of the 8169 men was 50.1 ± 15.2 years. The overall prevalence of MetS among this Chinese male population was 15.2%. The prevalence rates of current, former, and never smokers were 48.2% (3938/8196), 11.8% (961/8196), and 40.0% (3270/8196), respectively. The prevalence rates of current, former, and never drinkers were 46.5% (3796/8196), 7.0% (576/8196), and 46.5% (3797/8196), respectively. Participants with MetS were more likely to be older, have a higher BMI, exercise in their leisure time, and consume alcohol compared to men without MetS.

**Table 1.** Basic characteristics of participants by metabolic syndrome

Characteristics	MetS (*n* = 1243)	Non-MetS (*n* = 6926)	Total (*n* = 8169)
Age (years)^a^	51.4 ± 13.5	49.9 ± 15.5	50.1 ± 15.2
BMI (kg/m^2^)^a^	27.2 ± 2.5	22.5 ± 2.8	23.3 ± 3.2
Daily caloric intake (kcal)	2117.3 ± 642.2	2109.7 ± 642.0	2110.9 ± 642.0
Smoke status			
Nonsmoker	510 (41.0)	2760 (39.8)	3270 (40.0)
Ex-smoker	165 (13.3)	510 (11.5)	961 (11.8)
Current smoker	568 (45.7)	3370 (48.7)	3938 (48.2)
Drink status^b^			
Nondrinker	540 (43.4)	3256 (47.0)	3797 (46.5)
Ex-drinker	123 (9.9)	453 (6.6)	576 (7.0)
Current drinker	580 (46.7)	3217 (46.4)	3796 (46.5)
Smoking and alcohol intake^b^			
Neither	294 (23.7)	1799 (26.0)	2093 (25.6)
Only smoking	195 (15.7)	1202 (17.4)	1397 (17.1)
Only alcohol intake	184 (14.8)	875 (12.6)	1059 (13.0)
Both	325 (26.1)	2012 (29.0)	2337 (28.6)
Physical activity^b^			
Sedentary	994 (80.0)	6095 (88.0)	7089 (86.8)
Occasional exercisers	187 (15.0)	567 (8.2)	754 (9.2)
Conditioning exercisers	62 (5.0)	264 (3.8)	326 (4.0)
Metabolic syndrome	1243 (100.0)	—	1243 (15.2)
High waist circumference	1243 (100.0)	607 (8.8)	1850 (22.6)
High triglycerides	785 (63.2)	1384 (20.0)	2169 (26.6)
Low HDL-C	896 (72.1)	2704 (39.0)	3600 (44.1)
Hypertension	997 (80.2)	2781 (40.2)	3778 (46.2)
High fasting plasma glucose	545 (32.7)	1124 (16.2)	1669 (20.4)

BMI, body mass index; HDL-C, high-density lipoprotein cholesterol; MetS, metabolic syndrome.

Age and BMI are expressed as the mean ± standard deviation; other data are expressed as *n* (%).

^a^*P* < 0.001 comparing with Non-MetS by unpaired Student’s *t*-test.

^b^Pearson *P* < 0.05 by χ^2^-test.

Table 2 shows the number of patients and crude prevalence of MetS and its components stratified by cigarette smoking and alcohol consumption. The prevalence rates of MetS in nonsmokers, ex-smokers, and current smokers were 15.6%, 17.2%, and 14.4%, respectively. The prevalence rates of MetS in nondrinkers, ex-drinkers, and current drinkers were 14.2%, 21.4%, and 15.3%, respectively.

**Table 2.** Prevalence of metabolic syndrome and each component according to cigarette smoking and alcohol consumption status

Variable	Metabolic syndrome	Central obesity	High triglyceride	Low HDL-C	Hypertension	High fasting plasma glucose
Cigarette smoking status						
Nonsmoker	510 (15.6)	739 (22.6)	837 (25.6)	1461 (44.7)	1638 (41.6)	694 (21.2)
Ex-smoker	165 (17.2)	253 (26.3)	242 (25.2)	391 (40.7)	545 (56.8)	253 (26.3)
Current smoker	568 (14.4)	858 (21.8)	1090 (27.7)	1748 (44.4)	1595 (48.8)	722 (18.3)
Daily cigarette consumption (cigarettes/day)						
1–9	77 (15.7)	119 (24.2)	134 (27.3)	225 (45.8)	211 (43.0)	93 (18.9)
10–19	150 (14.4)	227 (21.7)	281 (26.9)	464 (44.4)	419 (40.1)	158 (15.1)
20–39	275 (13.4)	422 (20.6)	556 (27.1)	915 (44.7)	856 (41.8)	388 (18.9)
≥40	66 (18.6)	92 (26.0)	120 (33.9)	143 (40.4)	153 (43.2)	83 (23.4)
Alcohol consumption status						
Nondrinker	540 (14.2)	801 (21.1)	964 (25.4)	1913 (50.4)	1614 (42.5)	750 (19.8)
Ex-drinker	123 (21.4)	175 (30.4)	139 (24.1)	271 (47.0)	332 (57.6)	146 (25.3)
Current drinker	580 (15.3)	874 (23.0)	1066 (28.1)	1416 (37.3)	1832 (48.3)	773 (20.4)
Alcohol consumption (g/week)						
0.1–99	214 (18.2)	302 (25.6)	308 (26.1)	500 (42.4)	592 (50.2)	242 (20.5)
100–199	163 (15.5)	245 (23.3)	323 (30.7)	429 (40.8)	472 (45.0)	210 (20.0)
200–299	113 (13.6)	180 (21.7)	223 (26.9)	281 (33.9)	401 (48.4)	157 (18.9)
≥300	90 (12.2)	148 (20.1)	212 (28.8)	206 (28.0)	368 (49.9)	162 (22.0)

HDL-C, high-density lipoprotein cholesterol.

Data are reported as *n* (%).

### Relationship between cigarette smoking and prevalence of MetS and its components

No significant association was found between smoking status (either current or former smokers) and prevalent MetS, central obesity, or high TG. But current smoking was significantly associated with increased risk of low HDL-C and negatively associated with both hypertension and high fasting plasma glucose. After looking into the daily cigarette consumption among current smokers and adjusting for other risk factors, current smokers who smoked ≥40 cigarettes per day had a significantly higher risk of developing MetS (OR 1.34, 95% CI 1.01–1.79). Current daily cigarette consumption was significantly associated with the prevalence of high TG and low HDL-C. For high TG, current smokers who smoked ≥40 cigarettes per day had OR 1.50, while those with low HDL-C among all smokers, and in particular those who smoked 20–39 cigarettes, had significantly high OR. Further, the ORs for the prevalence of hypertension and high fasting plasma glucose were lower in current smokers than in nonsmokers (Table 3).

**Table 3.** Adjusted odds ratios of metabolic syndrome and its components in adult men according to cigarette smoking and alcohol consumption status

Variable	*n*	Metabolic syndrome	Central obesity	High triglyceride	Low HDL-C	Hypertension	High fasting plasma glucose
Cigarette smoking status							
Nonsmoker	3270	1.00	1.00	1.00	1.00	1.00	1.00
Ex-smoker	961	0.94 (0.76–1.15)	1.07 (0.90–1.29)	1.03 (0.87–1.23)	1.02 (0.87–1.19)	**0.79 (0.67–0.93)**^a^	0.99 (0.83–1.19)
Current smoker	3938	0.90 (0.78–1.03)	0.93 (0.83–1.05)	1.10 (0.98–1.23)	**1.21 (1.10–1.34)**^a^	**0.63 (0.57–0.70)**^a^	**0.83 (0.73–0.94)**^a^
Daily cigarette consumption (cigarettes/day)							
None	3270	1.00	1.00	1.00	1.00	1.00	1.00
1–9	491	0.94 (0.72–1.23)	1.00 (0.80–1.25)	1.05 (0.85–1.30)	1.21 (0.99–1.46)	**0.71 (0.58–0.89)**^a^	0.87 (0.68–1.11)
10–19	1045	0.89 (0.73–1.09)	0.90 (0.76–1.06)	1.03 (0.88–1.20)	1.11 (0.97–1.28)	**0.65 (0.56–0.75)**^a^	**0.66 (0.54–0.80)**^a^
20–39	2049	0.86 (0.74–1.01)	0.88 (0.76–1.00)	1.07 (0.94–1.22)	**1.27 (1.13–1.42)**^a^	**0.67 (0.59–0.75)**^a^	**0.87 (0.75–1.00)**
≥40	354	**1.34 (1.01–1.79)**	1.22 (0.95–1.57)	**1.50 (1.19–1.90)**^a^	1.13 (0.90–1.42)	**0.69 (0.55–0.87)**^a^	1.15 (0.88–1.49)
*P*_trend_		0.090	0.088	**0.007**	0.995	**<0.001**	**0.001**
Alcohol consumption status							
Nondrinker	3796	1.00	1.00	1.00	1.00	1.00	1.00
Ex-drinker	576	**1.60 (1.26–2.03)**^a^	**1.59 (1.29–1.96)**^a^	1.02 (0.81–1.24)	1.00 (0.83–1.20)	**1.30 (1.07–1.59)**	1.03 (0.82–1.28)
Current drinker	3797	1.14 (0.99–1.30)	**1.14 (1.02–1.28)**	**1.14 (1.03–1.27)**	**0.57 (0.52–0.63)**^a^	**1.35 (1.22–1.49)**^a^	1.05 (0.93–1.19)
Alcohol consumption (g/week)							
No alcohol	3796	1.00	1.00	1.00	1.00	1.00	1.00
0.1–99	1179	**1.22 (1.03–1.46)**	1.16 (0.99–1.36)	1.01 (0.87–1.18)	**0.70 (0.61–0.80)**^a^	**1.40 (1.20–1.59)**^a^	1.01 (0.85–1.19)
100–199	1051	1.05 (0.87–1.27)	1.08 (0.92–1.27)	**1.28 (1.10–1.49)**^a^	**0.65 (0.57–0.75)**^a^	**1.19 (1.03–1.38)**	1.07 (0.90–1.28)
200–299	829	0.90 (0.72–1.13)	1.01 (0.84–1.22)	1.11 (0.93–1.31)	**0.50 (0.42–0.58)**^a^	**1.28 (1.09–1.50)**^a^	0.95 (0.78–1.16)
≥300	737	0.84 (0.66–1.07)	0.98 (0.78–1.20)	**1.22 (1.02–1.47)**	**0.38 (0.32–0.46)**^a^	**1.34 (1.14–1.59)**^a^	1.14 (0.93–1.39)
*P*_trend_		0.050	0.332	**0.004**	**<0.001**	**0.003**	0.957

HDL-C, high-density lipoprotein cholesterol.

Data reported as odds ratio (95% confidence interval).

Odds ratios for cigarette smoking and metabolic syndrome and each component were calculated after adjustment for age, weekly alcohol consumption, physical activity, and daily total caloric intake.

Odds ratios for alcohol drinking and metabolic syndrome and its each component were calculated after adjustment for age, daily cigarette consumption, physical activity, and daily total caloric intake.

^a^The 95% confident intervals were adjusted under multiple comparisons.

### Relationship between alcohol consumption and prevalence of MetS and its components

Former alcohol consumption was significantly associated with increased prevalence of MetS (OR 1.60, 95% CI 1.26–2.03) and a marginally significant association of current drinking with MetS (OR 1.14, 95% CI 0.99–1.30) was found (Table 3). When looking into the drinking amount, adjusted OR of prevalent MetS in those who consumed 0.1–99 g per week was 1.22 (95% CI 1.03–1.46) compared to nondrinkers. A trend analysis showed an inverse relationship between alcohol intake and MetS (*P* = 0.05). Current alcohol consumption was significantly associated with increased central obesity, high TG, and hypertension. However, no dose-response relationship was found between alcohol consumption and central obesity. On the contrary, the OR of low HDL-C decreased as current alcohol consumption increased (*P*_trend_ < 0.001).

### Relationship between alcohol consumption and prevalence of MetS and its components according to cigarette consumption classification

Compared to those who neither drank nor smoked, those who were ex-smokers but nondrinkers, ex-drinkers but nonsmokers, both ex-drinkers and ex-smokers, ex-drinkers and current smokers, currents drinkers but nonsmokers, and current drinkers and ex-smokers had significantly high risk of developing central obesity (adjusted OR 1.49, 95% CI 1.10–2.01; 2.20, 95% CI 1.43–3.40; 1.47, 95% CI 1.05–2.05; 1.92, 95% CI 1.36–2.70; 1.32, 95% CI 1.09–1.59; and 1.38, 95% CI 1.05–1.82, respectively). The interaction of current smoking with former alcohol consumption was 1.67 (95% CI 1.24–2.25, *P* = 0.002) (Table 4). Nonsmoker, ex-smoker, and current smoker subgroups among current drinkers had higher risk of high TG than those who neither drank nor smoked (Table 5). Current drinkers but nonsmokers, current drinkers and ex-smokers, and both current drinkers and current smokers had lower prevalent low HDL-C than those who neither drank nor smoked. The interaction of current alcohol consumption with former smoking was 0.53 (95% CI 0.43–0.66, *P* < 0.001). The interaction of current alcohol consumption with current smoking was 0.66 (95% CI 0.60–0.73, *P* < 0.001) (Table 6). Current or former drinkers and nonsmokers had higher risk of developing hypertension (Table 7). When combining smoking and drinking status, the interactive OR of hypertension between current smoking and current drinking was 0.89 (95% CI 0.80–0.98). No interaction effect on prevalent high fasting plasma glucose was observed between smoking status and alcohol consumption status.

**Table 4.** Adjusted odds ratios of central obesity according to combined cigarette smoking and alcohol consumption status

	Nonsmoker	Ex-smoker	Current smoker
Nondrinker			
Prevalence (%)	20.7	25.8	20.7
Odds ratio	1.00	**1.49 (1.10–2.01)**	1.07 (0.90–1.28)
Ex-drinker			
Prevalence (%)	35.6	26.4	32.4
Odds ratio	**2.20 (1.43–3.40)**^a^	**1.47 (1.05–2.05)**	**1.92 (1.36–2.70)**^a,b^
Current drinker			
Prevalence (%)	25.0	26.7	21.5
Odds ratio	**1.32 (1.09–1.59)**	**1.38 (1.05–1.82)**	1.10 (0.93–1.27)

Data reported as odds ratios (95% confidence intervals), adjusted for age, daily total caloric intake, and physical activity.

^a^*P* value was considered significant using Bonferroni correction for multiple comparisons.

^b^Interaction = 1.67 (1.24–2.25), *P* = 0.002; reference was nondrinker and nonsmoker.

**Table 5.** Adjusted odds ratios of high triglyceride according to combined cigarette smoking and alcohol consumption status

	Nonsmoker	Ex-smoker	Current smoker
Nondrinker			
Prevalence (%)	24.2	24.8	27.3
Odds ratio	1.00	1.06 (0.77–1.44)	1.17 (0.99–1.38)
Ex-drinker			
Prevalence (%)	29.7	19.7	26.5
Odds ratio	1.40 (0.89–2.20)	0.92 (0.64–1.31)	1.30 (0.92–1.85)
Current drinker			
Prevalence (%)	28.0	28.9	28.0
Odds ratio	**1.21 (1.01–1.45)**	**1.34 (1.03–1.74)**	**1.26 (1.09–1.46)**^a^

Data are reported as odds ratios (95% confidence interval), adjusted for age, daily total caloric intake, and physical activity.

^a^*P* value was considered significant using Bonferroni correction for multiple comparisons.

**Table 6.** Adjusted odds ratios of low HDL-C levels according to combined cigarette smoking and alcohol consumption status

	Nonsmoker	Ex-smoker	Current smoker
Nondrinker			
Prevalence (%)	47.9	49.3	54.4
Odds ratio	1.00	1.17 (0.90–1.53)	**1.38 (1.19–1.59)**^a^
Ex-drinker			
Prevalence (%)	45.8	42.9	52.9
Odds ratio	1.04 (0.69–3.73)	0.99 (0.74–1.32)	**1.44 (1.05–1.97)**
Current drinker			
Prevalence (%)	38.2	32.7	37.7
Odds ratio	**0.68 (0.58–0.80)**^a^	**0.53 (0.41–0.68)**^a,b^	**0.69 (0.60–0.78)**^a,c^

Data are reported as odds ratios (95% confidence interval), adjusted for age, daily total caloric intake, and physical activity.

^a^*P* value was considered significant using Bonferroni correction for multiple comparisons.

^b^Interaction = 0.53 (0.43–0.66), *P* < 0.001; reference was nondrinker and nonsmoker.

^c^Interaction = 0.66 (0.60–0.73), *P* < 0.001; reference was nondrinker and nonsmoker.

**Table 7.** Adjusted odds ratios of hypertension according to combined cigarette smoking and alcohol consumption status

	Nonsmoker	Ex-smoker	Current smoker
Nondrinker			
Prevalence (%)	44.4	55.2	36.9
Odds ratio	1.00	1.27 (0.88–1.83)	1.11 (0.90–1.37)
Ex-drinker			
Prevalence (%)	69.5	55.5	53.4
Odds ratio	**2.32 (1.45–3.73)**^a^	0.75 (0.55–1.01)	0.82 (0.60–1.14)
Current drinker			
Prevalence (%)	55.1	58.8	43.4
Odds ratio	**1.37 (1.16–1.62)**^a^	1.18 (0.93–1.51)	**0.83 (0.73–0.95)**^b^

Data are reported as odds ratios (95% confidence interval), adjusted for age, daily total caloric intake, and physical activity.

^a^*P* value was considered significant using Bonferroni correction for multiple comparisons.

^b^Interaction = 0.89 (0.80–0.98), *P* = 0.024; reference was nondrinker and nonsmoker.

Compared with those who neither drank nor smoked, ex-drinkers who never smoked had significantly higher risk of developing MetS (adjusted OR 2.32, 95% CI 1.45–3.73); current drinkers who never smoked had an OR of 1.34 (95% CI 1.08–1.68); and those who were current smokers and ex-drinkers had an OR of 1.98 (95% CI 1.35–2.91). In addition, the interaction of current smoking with former alcohol consumption was 1.72 (95% CI 1.23–2.39, *P* = 0.001). Nevertheless, non-significant ORs of prevalent MetS were seen for all ex-smokers regardless of drinking status (Table 8). Bonferroni corrections for multiple tests revealed consistent results in most models.

**Table 8.** Adjusted odds ratios of metabolic syndrome according to combined cigarette smoking and alcohol consumption status

	Nonsmoker	Ex-smoker	Current smoker
Nondrinker			
Prevalence (%)	14.0	16.7	14.0
Odds ratio	1.00	1.27 (0.88–1.83)	1.11 (0.90–1.37)
Ex-drinker			
Prevalence (%)	27.1	16.9	23.5
Odds ratio	**2.32 (1.45–3.73)**^a^	1.11 (0.74–1.67)	**1.98 (1.35–2.91)**^a,b^
Current drinker			
Prevalence (%)	17.4	17.7	13.9
Odds ratio	**1.34 (1.08–1.68)**	1.38 (0.99–1.91)	1.10 (0.91–1.32)

Data are reported as odds ratios (95% confidence interval), adjusted for age, daily total caloric intake, and physical activity.

^a^*P* value was considered significant using bonferroni correction for multiple comparisons.

^b^Interaction = 1.72 (1.23–2.39), *P* = 0.001; reference was nondrinker and nonsmoker.

## DISCUSSION

After adjustment for age, alcohol intake, physical activity, and daily total calorie intake, heavy smokers (≥40 cigarettes/day) had a 34% increased risk of developing MetS compared with non-smokers. Our finding is in agreement with two studies conducted in Chinese men^5^^,^^23^ and several studies in other ethnic populations.^4^^,^^27^^–^^29^ However, our study did not find a dose-response relationship. We also found that mild consumption of alcohol (<99 g/week) in men was associated with a 22% increased prevalence of MetS compared to no alcohol consumption. And the lower odds ratio of prevalent MetS was related to increased alcohol consumption. Similarly, alcohol consumption has been shown to be inversely associated with MetS in a cohort study by a Norwegian group.^30^ After analyses of the components of MetS, our study indicated that increased daily amount of smoking was associated with decreased risk of hypertension and high plasma glucose but increased risk of high TG and low HDL-C. Increased weekly amount of alcohol consumption was associated with increased prevalence of hypertension and high TG and decreased prevalence of low HDL-C.

Our findings of smoking being associated with high TG and low HDL-C were consistent with previous studies.^4^^,^^5^ One possible explanation for this association is that nicotine may increase sympathetic nerve activity, which stimulates release of catecholamines and thereby induces lipolysis, with a consequent increase in plasma concentration of TG.^31^ Further, compared with nonsmokers, smokers have lower levels of the enzyme lecithin-cholesterol acyltransferase, which is involved in the cholesterol removal mediated by HDL-C, resulting in a reduction in plasma HDL-C levels.

Some epidemiological studies have found lower blood pressure in smokers than in nonsmokers.^32^^,^^33^ In the present study, we also found a negative association between cigarette smoking and blood pressure. The relationship between long-term smoking and hypertension is still unclear and controversial. It has been reported that smoking may lead to an acute increase in blood pressure due to release of catecholamines and vasopressin induced by nicotine.^28^ However, the measurement of blood pressure was often taken after a few hours’ gap in smoking, which generally reflected lower blood pressure than in nonsmokers.

Some population-based studies have reported that smoking increases the risk of diabetes,^27^^,^^34^ but one prospective study^23^ and several cross-sectional studies^35^^,^^36^ detected no such association. However, the odds ratio for high fasting plasma glucose was lower in current smokers than in nonsmokers in our study. The potential biological mechanism remains to be investigated in further studies.

No association between smoking and central obesity, indexed by high waist circumference, was found in Chinese men in our study or in other studies conducted in Chinese men.^5^^,^^23^^,^^37^ This finding is also consistent with a Norwegian study.^30^

Inconsistent results have been previously reported on the relationship between alcohol consumption and MetS. A cross-sectional study in a Japanese population found that alcohol consumption of more than 40.0 g per day was associated with increased prevalence of MetS.^38^ Gigleux et al^39^ reported that moderate alcohol consumers exhibited a more favorable metabolic profile than mild alcohol consumers. However, another study in Japanese men^28^ and a study in an elderly Italian population^40^ observed no relationship between alcohol consumption and MetS.

Reports on the relation of alcohol consumption with central obesity have been inconsistent.^8^^,^^41^^–^^43^ Our study detected higher risk of central obesity in current drinkers and former drinkers than nondrinkers. However, no dose-response relationship between central obesity and alcohol intake was observed.

Epidemiological data^21^^,^^44^ have shown that increased blood pressure is associated with increased alcohol consumption. These data are consistent with our findings that the odds ratio of hypertension increased as current alcohol consumption increased.

HDL-C has been shown to be higher in drinkers than in nondrinkers and tends to be higher as alcohol intake increases.^45^^,^^46^ Our present results are consistent with these previous findings. It is well known that HDL-C has protective effects on cardiovascular mortality. On the other hand, alcohol intake is also known to be related to elevated blood pressure and TG levels^47^ and higher waist circumference. Further research is needed to determine the net benefit or harm of alcohol intake.

Overall, modest associations between cigarette smoking and alcohol consumption and MetS as a whole were found after we looked into the categorical amounts of smoking and drinking in our study. Neither current smoking nor current drinking status was initially statistically associated with MetS. The relationship between cigarette smoking/alcohol consumption and MetS greatly depends on the prevalence of its individual components. Assessing the influence of smoking and drinking on MetS has much larger public health implications than assessing one component since the five conditions that make up MetS are often diagnosed in the same person.

A noteworthy result from our study is that ex-drinkers had higher adjusted ORs of MetS and central obesity than in non-drinkers. This finding was consistent with that in an Irish study.^17^ Former drinkers may have stopped their drinking in response to poor health, possibly explaining why ORs of MetS were significantly increased for smoking and nonsmoking ex-drinkers but not for nondrinking ex-smokers.

Another noteworthy finding is that the combination of being a non-smoker and current/former alcohol consumption was significantly associated with the prevalence of most components of MetS and MetS as a whole. However, no association was detected for the combination of non-drinker and current smoker/former smoker. In our study, drinking may play a more important role than smoking in the development of MetS. It is known that smoking and alcohol consumption are highly correlated. Interaction of current smoking with former alcohol consumption on the increased prevalence of MetS and central obesity was observed. And current smoking interacted with former alcohol consumption to decrease the risk of developing low HDL-C. However, the detailed mechanism behind this association is unknown. Nakashita et al^4^ showed that combined current smoking and drinking was associated with MetS. However, no interactive effect of smoking and drinking was reported by Takeuchi^28^ or by Cai.^48^

Our study has a few strengths. Our study was the first to examine the interactive effects of cigarette smoking and alcohol consumption on MetS in China and includes a large number of participants. Considering the limited number of studies conducted in Chinese population, this study is a great addition to the previous literature.

Our study has some limitations. First, this is a cross-sectional study, which limits causal inferences. Second, there might be some recall bias, particularly concerning the number of cigarettes smoked per day among ex-smokers and amount of alcohol consumed per week among ex-drinkers. Third, the information regarding cigarette smoking and alcohol consumption was self-reported. Thus, potential misclassification may have occurred in some cases, but it should be random or non-differential among the groups.

In conclusion, this cross-sectional study indicated that cigarette smoking and alcohol consumption are independently associated with MetS. We observed interactions of smoking and drinking on risk of MetS among men in China. However, these associations need to be confirmed in future large-scale longitudinal studies.
